# Reliability of a Measurement Method for Upper Limb Raised Standing Spinal Alignment Using a Smartphone Inclinometer Application

**DOI:** 10.7759/cureus.76225

**Published:** 2024-12-22

**Authors:** Hiroki Takayama, Koji Miyawaki, Masatoshi Nakamura

**Affiliations:** 1 Department of Physical Therapy, Hanna Central College of Rehabilitation, Shijonawate, JPN; 2 Department of Physical Therapy, Faculty of Rehabilitation Sciences, Nishikyushu University, Saga, JPN

**Keywords:** inclinometer, lumbar lordosis, overhead position, smartphone, thoracic kyphosis

## Abstract

Purpose: To evaluate the reliability and validity of spinal alignment measurements in the raised arm standing posture using a smartphone app.

Background: An inclinometer is a reliable tool for measuring spinal alignment. Measurement of static standing posture spinal curvature angles using smartphone inclinometer applications has been investigated in the lumbar spine but has not been reported for the thoracic spine. However, the sacral vertebrae were used as the reference point for the measurement of the lumbar spine, and the method of palpation of the sacral vertebrae was unclear. No measurement methods, including inclinometers, have been found for upper limb elevation.

Methods: Thoracic kyphosis and lumbar lordosis angles were measured in 18 healthy young adults (mean age: 21.0 ± 3.5). Measurements were taken in the raised standing posture. The points of measurement included angle α at the thoracic spine (T)1/2, angle β at the T12/lumbar spine (L)1, and angle γ at the L4/L5 spinous processes. The thoracic kyphosis angle was calculated as the sum of α and β, while the lumbar lordosis angle was the sum of β and γ. Two raters measured these angles twice using the same smartphones. Inter-rater reliability was assessed using intraclass correlation coefficients (ICC 2.1), and measurement precision was determined by calculating MDC95 from the ICC values. Validity was also carried out on 12 healthy young adults (age: 20.8 ± 4.0 years). The measurement points were the same as described above, and two types of measurements were taken with a smartphone and an inclinometer; the results of the two types of measurements were used to determine the relationship using Pearson's correlation coefficient.

Results: During upper limb raising, the smartphone’s ICC (95% confidence interval) was 0.92 (0.81-0.97) for thoracic kyphosis and 0.90 (0.74-0.96) for lumbar lordosis. The MDC95 values indicated acceptable precision, with 7.00° for thoracic kyphosis and 9.95° for lumbar lordosis. All correlation coefficients between inclinometers and smartphones were above 0.9 for both the thoracic and lumbar spines.

Conclusions: Measuring spinal alignment angles using a smartphone inclinometer app in the raised standing posture demonstrates good reliability for inter-rater comparisons. It was also good with regard to validity.

## Introduction

Postural observation and analysis are common in physical therapy and sports settings, especially when working with athletes. Spinal alignment, such as thoracic kyphosis and lumbar lordosis, is often assessed in these cases. Studies have shown that spinal alignment is correlated to shoulder and lumbar injuries [1,2]. Therefore, regular assessments are essential to prevent shoulder and back injuries, even in the absence of symptoms. However, physiotherapists and athletic trainers often rely on subjective assessments. The most commonly used objective methods for assessing spinal alignment include X-rays and three-dimensional motion analysis systems. These tools, however, are expensive and impractical for daily use in the field, and X-rays raise concerns regarding radiation exposure. A more cost-effective and objective approach is to utilize an inclinometer or a smartphone application that includes an inclinometer function. Inclinometer-based assessments of thoracic kyphosis in a seated position are highly reproducible [3] and have been shown to correlate well with X-ray assessments of thoracic kyphosis in the standing position [4-6]. Conversely, there are reports of smartphone inclinometer applications being used to assess lumbar spine movements, such as anterior flexion and backward bending, in the standing position [7]. As of 2012, the adoption of smartphones among physicians was estimated at 82% [8], a figure that has likely increased since then. Smartphone applications in medical practice offer convenience for both education and clinical decision-making [9]. Additionally, they are quick and easy to use, given the widespread availability of smartphones. We utilized a smartphone inclinometer application to measure spinal alignment in a standing position with raised upper limbs. In sports such as swimming and volleyball, athletes frequently raise both arms, making this posture relevant for assessing spinal alignment. Smartphone measurements of thoracic kyphosis and lumbar lordosis may aid in preventing injuries and enhancing performance. However, there is currently no data regarding the reliability and validity of using smartphones to measure these angles. Additionally, it is uncertain how spinal curvature angles can be assessed in an upright position with raised arms, miming sports movements using an inclinometer.

This study has two objectives: first, to assess the reliability of smartphone measurements for thoracic kyphosis and lumbar lordosis angles in relaxed and raised arm positions while standing and, second, to validate these smartphone measurements by comparing them with standard inclinometer assessments.

## Materials and methods

Participants

Eighteen healthy young adults (nine males and nine females; age: 21.0 ± 3.5 years; height: 163.8 ± 7.1 cm; weight: 59.4 ± 11.8 kg) participated in the reliability study (Study 1). This sample size was determined based on a 5% significance level and 80% power (b = 0.20), with a true p0 of 0.7 versus an alternative p1 of 0.9 for two raters or time points, requiring 18.4 participants, which was rounded to 18 [10]. Individuals with a history of shoulder or spinal injuries or those unable to hold 180° shoulder flexion were excluded. The presence or absence of a pre-existing injury was assumed to have been diagnosed by a doctor. In addition, the lumbar lordosis is allowed to increase during upper limb elevation. For the validation study (Study 2), 12 healthy adults (six males and six females; age: 20.8 ± 4.0 years; height: 164.7 ± 7.5 cm; weight: 62.4 ± 12.5 kg) participated, using the same inclusion and exclusion criteria as in Study 1. The number of participants was determined using G*Power (version 3.1; The G*Power Team, Germany), with an effect size of 0.7, a 5% significance level, and 80% power (b = 0.20), requiring 11 participants. However, 12 were included to ensure equal gender representation. The measurements were taken between 15 March and 1 July 2023. All participants in both Studies 1 and 2 were Japanese. This is because the participants who offered to participate in the studies were only Japanese. The study received approval from the Hanna Central College of Rehabilitation Ethics Committee (Date. 22 February 2023/No. HCCR-002).

Data collection

The tools utilized for measurement were an inclinometer (ICHINEN AXESS Corporation, 1-1-6 Sumiyoshi, Ikeda, Osaka, Japan) and the built-in “Measure” application on the iPhone SE2® (Apple, One Apple Park Way, Cupertino, CA). The “Measure” application utilizes the built-in accelerometer of iPhone® and a digital display to indicate the measured angle. The accuracy of the inclinometers and the applications used were not reported by the respective manufacturers. Three preliminary measurements were conducted on a level surface prior to the start of the main measurements, and all confirmed the same values. Two physiotherapists were involved in evaluating the reliability of the smartphone: Rater A, an experienced professional with 12 years of clinical practice, and Rater B, a first-time user with eight years of clinical experience. Rater B underwent a single orientation session and practiced beforehand. Rater A performed the validation using both the inclinometer and the smartphone for comparison.

Measurement protocol

**Figure 1:**
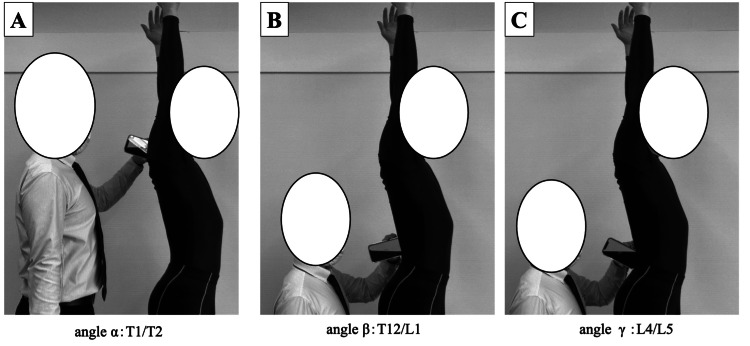
Measuring landmarks of spinal alignment A. angle α: T1/T2   B. angle β: T12/L1   C. angle γ: L4/L5 After identifying each point, the angles were measured using the inclinometer and smartphone, with the device positioned so that the center of the short side was between the spinous processes. This image is original to this study.

The participants were measured in two postures: a relaxed standing position with their arms at the side and a raised position with the shoulder joint flexed at 180º. Bilateral shoulder flexion of 180° was confirmed by the measurer and his assistant using a goniometer. The assistants monitored for compensatory movements of the thoracic and lumbar spine during the measurements. They wore tight-fitting compression garments for accurate body contact. The measurement points included angle α at the thoracic spine (T)1/2, angle β at the T12/lumbar spine (L)1, and angle γ at the spinous processes of L4/L5. Palpation was done without the use of markers, simulating real clinical settings. Participants were instructed to achieve a natural posture by swinging their arms three times, stopping in a comfortable position, bending and stretching their heads three times, and breathing deeply three times before assuming a natural posture [3]. The method for measuring angles is shown in Figure 1. For angle α, the prominent spinous process of the seventh cervical vertebra was identified [11]. T1 was located by asking the participant to rotate their neck. T1 was the first superior spinous process that remained stationary during rotation, with T2 immediately below. For angles β and γ, the L3/L4 interspinous space was identified using the Jacoby line connecting the highest points of the iliac crests [12]. After verifying the sacrum, the spinous processes were traced upward to locate T12/L1 and L4/L5. Once identified, the angles were measured using the inclinometer and smartphone, with the device positioned so that the center of the short side was between the spinous processes. The instrument was held for five seconds to allow the measurement values to stabilize. The absolute values displayed on the devices were recorded.

**Figure 2:**
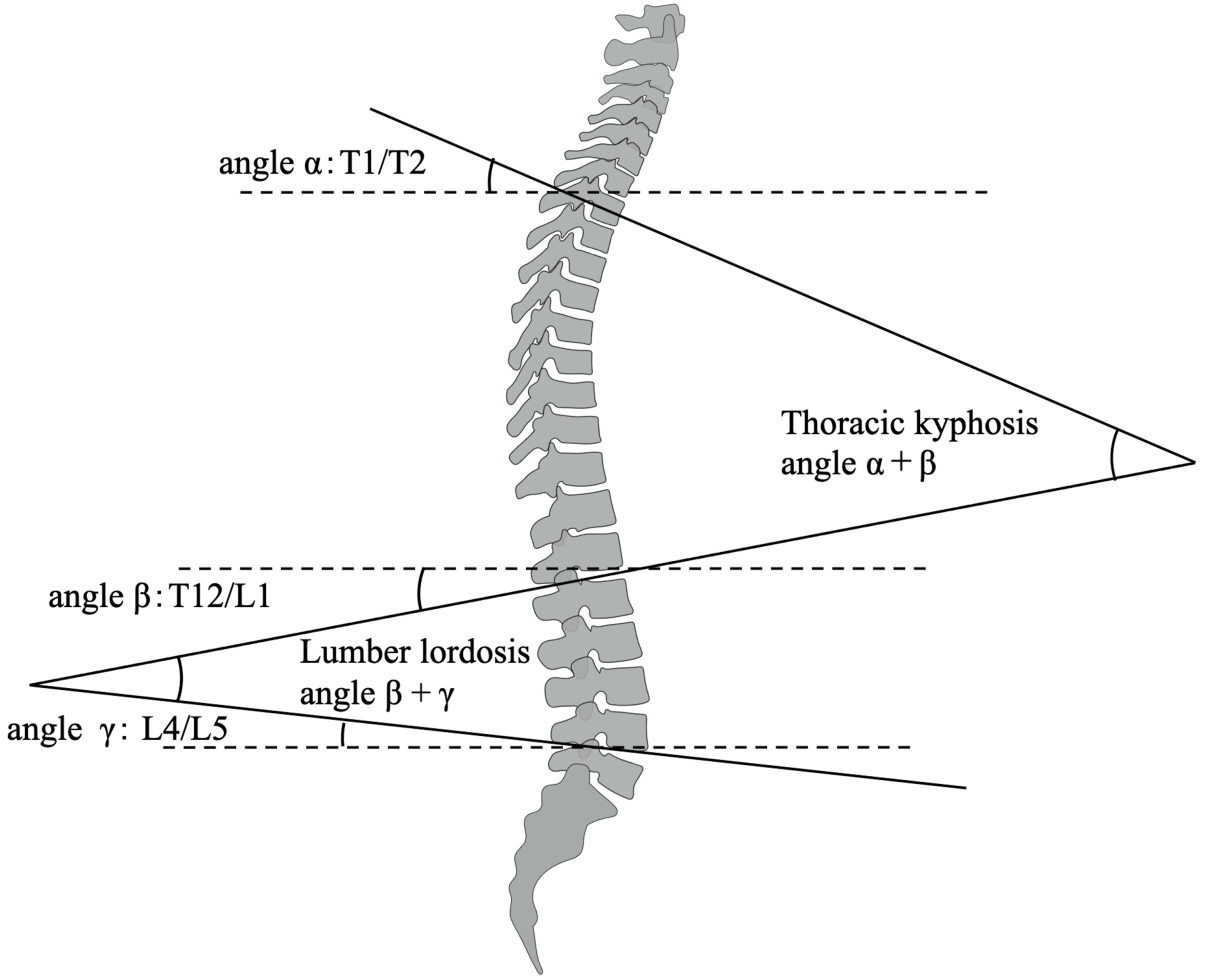
How to calculate the thoracic kyphosis angle and lumbar lordosis angle This image is original to this study.

The calculation of the thoracic kyphosis angle and lumbar lordosis angle is shown in Figure 2. The thoracic kyphosis angle is determined by summing angles α and β, while the lumbar lordosis angle is the sum of angles β and γ. The calculation of the thoracic kyphosis angle is based on previous research [3,5]. Traditionally, the lumbar lordosis angle is calculated using the tilt angle between the upper first lumbar vertebra and the sacral base [13]. However, since the prior study did not detail how to palpate the sacral landmarks, this study utilized the angle between L4/L5.

For reliability verification, Rater A and Rater B each took measurements twice. Rater A conducted the first measurement in both the relaxed and raised upper limb positions, followed by Rater B, who performed the second without knowing Rater A’s results to maintain blinding. After a rest period of approximately three hours, the second measurement was taken to ensure at least a 30-minute gap, reducing examiner bias and mitigating memory recall issues from the first measurement [3]. Each rater verbally reported their findings to an assistant, who recorded them on a designated assessment sheet. For validation, measurements were initially taken with an inclinometer and repeated three hours later with a smartphone.

Statistical analysis

Statistical analysis was conducted using EZR (version 1.64) [14]. This software is distributed free of charge on the website of the Department of Haematology, Saitama Medical Centre, Jichi Medical University, Saitama, Japan. The normality of all measurement data was assessed with the Shapiro-Wilk test. The reliability of smartphone measurements was assessed using both relative and absolute reliability methods. Relative reliability was determined through intraclass correlation coefficients (ICC). Specifically, ICC (1,1) was calculated for the first and second measurements by Rater A, while ICC (2,1) was used for interclass reliability based on the initial measurements. The 95% confidence intervals (95% CI) were computed. Reliability values were classified based on the lower limit of each 95% CI, following established criteria (<0.50 poor, 0.50-0.75 moderate, 0.75-0.90 good, >0.90 excellent) [15].

Absolute reliability was evaluated using the standard error of measurement (SEM) to determine the accuracy of the measurement device. The SEM formula is derived from the “standard deviation of the measured value group x √1-ICC” [16]. The SEM and the minimum detectable change at the 95% CI (MDC95) were calculated [16,17]. MDC95 indicates the minimum change needed to be confident that an observed change is not due to random variation or measurement error. The MDC95 is calculated using the formula “SEM × 95% CI z-value (1.96) × √2” [17]. Bland-Altman analysis was applied to calculate the SEM and MDC95, ensuring that no additive nor proportional errors were present.

To validate the smartphone measurements, the correlation coefficient between the thoracic kyphosis and lumbar lordosis angles was computed using the Pearson correlation coefficient. Student t-tests were conducted to identify significant differences in the measurements from each instrument. The normality of the data was checked with the Shapiro-Wilk test, and equal variance was assessed using the F-test.

## Results

ICC, SEM, and MDC95 of smartphone measurements

The results of thoracic kyphosis measurements are shown in Table 1, and lumbar lordosis measurements in Table 2.

**Table 1:** Measurement results of the thoracic kyphosis angle ICC: Intraclass correlation coefficients; SEM: Standard error of measurement; MDC95: Minimum detectable change at the 95%CI The ICC of A is (1,1), and the ICC of inter-rater reliability is (2,1).

		mean ± SD（°）	ICC (95%CI)	SEM	MDC95
A	Relax upper limb	35.5 ± 10.1	0.91 (0.78-0.97)	2.5	6.9
	Raise upper limb	26.9 ± 9.8	0.96 (0.90-0.99)	1.7	4.8
Inter-rater reliability	Relax upper limb	36.1 ± 10.4	0.90 (0.76-0.96)	2.5	7.1
	Raise upper limb	26.8 ± 7.0	0.92 (0.81-0.97)	2.5	7.0

**Table 2:** Measurement results of the lumbar lordosis angle ICC: Intraclass correlation coefficients　SEM: Standard error of measurement MDC95: Minimum detectable change at the 95%CI The ICC of A is (1,1) and the ICC of Inter-rater reliability is (2,1).

		mean ± SD（°）	ICC (95%CI)	SEM	MDC95
A	Relax upper limb	25.6 ± 10.4	0.91 (0.77-0.96)	2.6	7.4
	Raise upper limb	26.7 ± 12.2	0.98 (0.95-0.99)	1.6	4.4
Inter-rater reliability	Relax upper limb	25.8 ± 9.2	0.88 (0.71-0.95)	2.8	7.9
	Raise upper limb	26.5 ± 11.9	0.90 (0.74-0.96)	3.5	9.9

Results for thoracic kyphosis angle measurements are shown in Table 1. For thoracic kyphosis with relaxed upper limbs, ICC (1,1) was 0.91, and ICC (2,1) was 0.90. For lumbar lordosis, ICC (1,1) was 0.91, and ICC (2,1) was 0.88. When upper limbs were raised, thoracic kyphosis ICC (1,1) increased to 0.96 and ICC (2,1) to 0.92. Lumbar lordosis measurements showed ICC (1,1) of 0.98 and ICC (2,1) of 0.90.

The SEM for thoracic kyphosis with relaxed limbs was 2.5 for Rater A and 2.5 between A and B. For lumbar lordosis, SEM was 2.6 for A and 2.8 between A and B. In the raised position, thoracic kyphosis SEM was 1.7 for A and 2.5 between A and B; lumbar lordosis had an SEM of 1.6 for A and 3.5 between A and B.

MDC95 for thoracic kyphosis with relaxed limbs was 6.9 for A and 7.1 between A and B. For lumbar lordosis, MDC95 was 7.4 for A and 7.9 between A and B. When limbs were raised, thoracic kyphosis MDC95 was 4.8 for A and 7.0 between A and B; lumbar lordosis was 4.4 for A and 9.9 between A and B.

Validity of smartphone measurements

The results of the comparison of inclinometer and smartphone measurements are shown in Table 3. No significant differences were observed between inclinometer and smartphone measurements.

**Table 3:** Student's t-test smartphone vs inclinometer

	Thoracic kyphosis (°)		Lumbar lordosis (°)	
	Relax upper limb	Raise upper limb	Relax upper limb	Raise upper limb
Smartphone	35.5 ± 8.4	28.0 ± 9.4	22.8 ± 8.3	27.9 ± 10.0
inclinometer	38.2 ± 8.3	27.4 ± 9.8	22.4 ± 8.9	27.9 ± 10.5
p value	0.846	0.884	0.908	0.891

**Figure 3:**
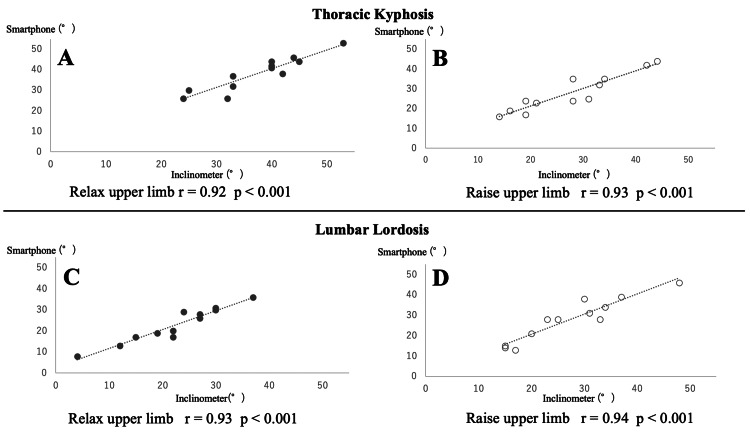
Correlation between the smartphone and inclinometer measurement A. Thoracic kyphosis with upper limb relaxed; B. Thoracic kyphosis with upper limb raises; C. Lumbar lordosis with upper limbs relaxed; D. Lumbar lordosis with upper limb raises

Figure 3 shows the correlation between the inclinometer and smartphone measurements. Both thoracic kyphosis and lumbar lordosis measurements correlated above 0.9, regardless of upper limb position.

## Discussion

This study evaluated the reliability and validity of using a smartphone to measure thoracic kyphosis and lumbar lordosis angles. A key aspect of this research is its focus on measurements taken in a standing position with the upper limbs raised. Previous studies have indicated a link between the thoracic kyphosis angle and shoulder joint injuries, as well as the maximum range of motion of the shoulder joint [18]. While scapulothoracic joint mobility can be assessed using a stick for backward rotation, diving athletes have reported that limited mobility in this joint may increase the risk of lower back pain [19]. Additionally, another study found that athletes exhibiting greater lumbar lordosis during dolphin kicks in swimming are more likely to experience low back pain [20]. Therefore, it is crucial for physical therapists and athletic trainers to have a reliable and easy method to measure thoracic kyphosis and lumbar lordosis angles during upper limb raising using commonly available smartphones.

The lower limits of the 95% CI for smartphone measurements of the thoracic kyphosis angle in the relaxed upper limb position were ICC (1,1) of 0.78 and ICC (2,1) of 0.76. For the upper limb elevated position, the lower limits were ICC (1,1) of 0.90 and ICC (2,1) of 0.81. These results indicate “good” measurement reproducibility [15]. Previous studies have also reported high ICC values for thoracic kyphosis angle measurements in the relaxed position [3,4]. The enhanced reliability of these measurements may be due to the secure placement of the device, which minimized slippage during movement and facilitated accurate identification of the spinous processes. Thus, measuring the thoracic kyphosis angle with a smartphone is considered a relatively reliable method.

The lower limits of the 95% CI for lumbar lordosis angle measurements on the smartphone were ICC (1,1) of 0.77 and ICC (2,1) of 0.71. For the upper limb elevated position, the lower limits were ICC (1,1) of 0.95 and ICC (2,1) of 0.74. These results indicate “moderate” measurement reproducibility for lumbar lordosis [15]. The lower reproducibility compared to thoracic kyphosis measurements may stem from the challenges in palpating the L4/L5 spinous processes. Even experienced physical therapists and chiropractors have shown inconsistency in palpating these processes [21,22]. Additionally, factors such as the thickness of the participant's soft tissue and the height of the spinous processes can impact palpation accuracy [20]. It is important to note that an inexperienced person measuring lumbar lordosis may be less reliable due to inadequate palpation skills and variations in participants' body sizes. Some studies have identified the landmarks for angle γ as the L5/sacral vertebra (S)1, S1/S2, and S2/S3 [23-25]. ICC (2,1) is better when the landmarks are restricted to the pelvic region [22,23]. Thus, using bony references in the sacral area may enhance reproducibility. However, previous studies have shortcomings as they do not clearly outline how to identify each of the sacral landmarks. Clarifying these landmarks could lead to a more reliable method for measuring the lumbar lordosis angle. Additionally, the proficiency of the person measuring the lumbar lordosis angle may affect the results.

In a previous study, the intraexaminer SEM for thoracic kyphosis angle measurement in the relaxed upper limb position was reported as 2.4° [3] for the inclinometer, while the interexaminer SEM was 2.3° [23]. No reports were found on MDC95. Rater A’s SEMs were similar to or lower in both relaxed and raised positions. The SEM formula includes ICC [16], and a higher ICC results in a lower SEM. Therefore, the good ICC for thoracic kyphosis measurement using a smartphone corresponds with SEM values similar to those in previous studies. Measurements within the MDC95 range are attributed to measurement error, while changes exceeding this range are considered “true changes” with a 5% risk of error [17]. Thus, the measurement error range for thoracic kyphosis angle measurement may be approximately 4.89°-7.15°, and changes beyond this range are regarded as true changes.

In a previous study on lumbar lordosis angle measurements using a smartphone, the interexaminer SEM was found to be 2.13°, and MDC95 was 5.9° [25]. In the current study, the SEM for lumbar lordosis measurements was significantly higher, regardless of whether the upper limb was raised. This indicates that a higher SEM corresponds to a higher MDC95, while a lower ICC also results in a higher MDC95, as the ICC value influences the SEM. Previous studies have employed methods to enhance reliability, such as marking the skin directly [23-25]. Other strategies, such as allowing the rater to practice beforehand, have also been utilized [24]. However, these methods are impractical in clinical or sports settings due to the required preparation time and skin exposure. The current method is more practical, relying solely on palpation without removing clothing. Nevertheless, The large MDC95 measurement error suggests that this method may not be suitable for assessing lumbar lordosis angles. However, measuring thoracic kyphosis angles, including upper limb elevation, may still be beneficial.

Previous studies have demonstrated a positive correlation (r = 0.62-0.86) between X-ray measurements and thoracic kyphosis angles obtained with an inclinometer [5,6]. Additionally, a strong positive correlation (r = 0.7 or higher) was found between X-ray and telemetry measurements for lumbar lordosis and thoracic kyphosis range of motion at similar points (T12, L1) as in the current study [26]. These findings suggest that using an inclinometer to measure thoracic kyphosis angles is a straightforward, quick, and valid approach. The correlation between smartphone and inclinometer measurements for lumbar lordosis angles was reported as r = 0.86 [24], and for thoracic rotation angles measured on a smartphone, it was r = 0.98 [27]. This indicates that smartphone measurements are also valid, similar to inclinometer measurements. Both smartphone and inclinometer measurements of thoracic kyphosis and lumbar lordosis showed correlations above 0.9, with no significant differences between the methods, regardless of upper limb elevation. These results imply that the smartphone measurement method is as valid as the inclinometer method.

Limitation

The participants in this study were young individuals, so the results may differ for older adults. Additionally, the skill level of the examiner might have varied between the first and 18th measurements. This measurement did not correlate with established methods such as X-rays or 3D motion analysis, and finding such correlations would have strengthened the validity of this approach.

## Conclusions

The reliability and validity of using a smartphone to measure thoracic kyphosis and lumbar lordosis angles were confirmed. Thoracic kyphosis angle measurements were reliable regardless of upper limb position, while lumbar lordosis reliability varied with the rater’s skill level. Smartphone measurements were validated against inclinometers. There was no significant difference between the measurements from the smartphone and the inclinometer, and a high positive correlation was found. These results suggest that this method can efficiently and accurately assess spinal curvature angles in sports and physiotherapy contexts.
